# Facile preparation of water-soluble hyperbranched polyamine functionalized multiwalled carbon nanotubes for high-efficiency organic dye removal from aqueous solution

**DOI:** 10.1038/s41598-017-03490-6

**Published:** 2017-06-15

**Authors:** Lihua Hu, Zhongping Yang, Yaoguang Wang, Yan Li, Dawei Fan, Di Wu, Qin Wei, Bin Du

**Affiliations:** 1 Key Laboratory of Chemical Sensing & Analysis in Universities of Shandong, School of Chemistry and Chemical Engineering, University of Jinan, Jinan, 250022 PR China; 2 School of Resources and Environment, University of Jinan, Jinan, 250022 PR China

## Abstract

Water-soluble hyperbranched polyamine functionalized multiwalled carbon nanotubes nanocomposite (WHPA-OMCNT) was successfully prepared and applied to water remediation in this paper. WHPA-OMCNT was characterized by Fourier transform infrared spectroscopy (FTIR), thermogravimetric analysis (TGA), zeta potential, scanning electron microscopy (SEM) and transmission electron microscope (TEM) analyses. WHPA-OMCNT exhibited excellent adsorption performance for removal of organic dyes e.g., methylene blue (MB), malachite green (MG) and methyl violet (MV). The equilibrium adsorption capacity was 800.0 mg g^−1^ for MB, 840.3 mg g^−1^ for MG and 970.9 mg g^−1^ for MV under the optimal conditions. The pseudo-second order equation and the Langmuir model exhibited good correlation with the adsorption kinetic and isotherm data for all three pollutants, respectively. The thermodynamic results (Δ*G* < 0, Δ*H* < 0, Δ*S* < 0) implied that the adsorption process of MB, MG and MV was feasible, exothermic and spontaneous in nature. A possible adsorption mechanism has been proposed, where H-bonding, electrostatic attraction and π-π stacking interactions dominated the adsorption of the organic dyes. In addition, the excellent reproducibility endowed WHPA-OMCNT with the potential for application in water treatment.

## Introduction

For years, water pollution with toxic organic dyes from developing modern industries, such as textile, plastic, leather, cosmetics, paper, and food, has been a serious environmental issue. The colored wastewater discharged from these industries may create an eco-toxic hazard and cause bioaccumulation. Therefore, the decontamination of wastewater containing toxic organic dyes has received a great deal of attention^1^. Dyes with a complex aromatic structure are very stable and difficult to be biodegraded. Methylene blue (MB), malachite green (MG), and methyl violet (MV), which are the most commonly used substances for dying silk, wool, cotton or wood, may cause neurological injury, nausea and vomiting^2^. Due to the strong toxicity, nonbiodegradability and accumulation in plants, animals and human beings, organic dyes should be cleaned from wastewater prior to being released into the environment. Numerous chemical, physical and biological treatment methods have been developed to treat the dye-containing wastewater in recent years^3^. Among these methods, the adsorption technique has proven to be an effective, simple, and economically feasible method for removal of organic dyes from wastewater^4, 5^. Therefore, various adsorbents, such as activated carbon^6^, zeolite^7^, clay^8^ and sepiolite^9^, have been produced to meet different needs. However, these traditional adsorbents are typically limited by low adsorption capacities or recycling problems. Therefore, the development of new adsorbents with better adsorption performance is highly desirable.

Recently, nanostructured materials with high surface area and high activities caused by the size-quantization effect have been widely explored as high-efficiency sorbents. As a key member of the carbon family, carbon nanotubes (CNT) with a nano-sized diameter and tubular microstructure have been widely researched in various fields such as electronic devices, catalysis, drug delivery and composite hydrogen storage^10, 11^. Furthermore, because of the excellent structural features and high surface areas, CNT have shown a strong binding affinity with heavy metal ions and organic dyes through the combination of hydrophobic, electrostatic, and π-π stacking interactions^12–14^. More importantly, compared with traditional adsorbents, such as activated carbon, research found that CNT showed a higher efficiency in removing pollutants in wastewater because of their unique structure and large surface areas. However, the adsorption performance of CNT is limited by the relatively low density of surface functional groups and poor water dispersibility caused by aggregation via the strong intertube π-π stacking interactions. To improve the dispersion properties in aqueous solutions as well as the adsorption capacity, CNT are often functionalized with inorganic nanoparticles, surfactants, hydrophilic groups, or polymers^15–21^.

It is known that functional groups, such as hydroxyl, amine, thiol and carbonyl, have strong interactions with various synthetic dyes^22^. Therefore, water-soluble polymers containing desired functional groups are typically chosen to modify CNT for improving dispersion properties in aqueous solutions as well as the adsorption capacity. For example, Gao *et al*.^23^ applied ionic liquid-based polyether to modify CNT to prepare a novel adsorbent with high adsorption capacities for anionic azo dyes. Chatterjee *et al*.^24^ successfully prepared chitosan hydrogel beads generated by sodium dodecyl sulfate gelation with multiwalled carbon nanotubes impregnation as a new adsorbent for the removal of Congo red from aqueous solution. Xie *et al*.^25^ fabricated a poly(sodium-p-styrene sulfonate) modified multiwalled carbon nanotubes composite, which was used for the removal of methyl blue with a high capacity.

Compared with traditional linear polymers, hyperbranched polymers (HPs) are more suitable for the modification of CNT to prepare novel adsorbents due to their nearly spherical structure and numerous surface functional groups. Moreover, the functional groups are generally located on the surface of the hyperbranched polymer due to weak or even non-existent molecular chain entanglements that present in HP, so most of the functional groups are in close contact with adsorbates^26^. Recently, researchers reported that HP modified substrates, such as magnetic particles^27^, collagen fibers^28^, silica-gels^29^ and graphene^30^, realized effective removal of organic dyes and heavy metals. However, as far as we know, few studies have employed water-soluble hyperbranched polyamine functionalized CNT as an adsorbent.

In this study, we combined water-soluble hyperbranched polyamine (WHPA) with high surface area oxidized multiwalled carbon nanotubes (OMCNT) to prepare a novel nanosorbent (WHPA-OMCNT) for the high removal efficiency of organic dyes. The obtained WHPA-OMCNT exhibited good water solubility and strong affinity toward the dyes due to the existence of abundant hydroxyl, ether and amine groups. Therefore, anchoring of WHPA onto the CNT surface is beneficial for improving the adsorption capacities of dyes. The synthesized WHPA-OMCNT was characterized by FTIR, TGA, zeta potential, SEM and TEM analyses. Batch adsorption tests of MB, MG and MV by WHPA-OMCNT were carried out to study the adsorption kinetics, isotherms and thermodynamics, and a possible adsorption mechanism was also accordingly proposed. In addition, to further evaluate the practical applications, the effect of coexisting ions on the adsorption and the regeneration performance of WHPA-OMCNT were also investigated.

## Materials and Methods

### Chemicals and materials

All reagents used in the experiment were of analytical reagent grade. Multiwalled carbon nanotubes (MCNT, Chengdu Organic Chemicals Co. Ltd., Chinese Academy of Sciences, China), Poly(ethylene oxide) diglycidyl ether (PEO-DGE, Sigma Aldrich, USA) and N-ethylethylene diamine (EEDA, Alfa Aesar, London) were used as received. 1-Ethyl-3-(3-dimethylaminopropyl) carbodiimide hydrochloride (EDC), N-hydroxyl succinimide (NHS) and other chemicals were obtained from Sinopharm Chemical Reagent Beijing Co., Ltd, China. In addition, methylene blue, malachite green and methylene violet, which were employed as the dye source, were dissolved in ultrapure water prior to use. For pH adjustment, 0.1 mol L^−1^ HCl and 0.1 mol L^−1^ NaOH were used.

### Synthesis of water-soluble hyperbranched polyamine (WHPA)

WHPA was synthesized through the nucleophilic ring-opening reaction of diepoxy and diamine monomer^31^. The reaction was conducted in a three-neck flask equipped with a nitrogen inlet tube and a reflux condenser. PEO-DGE (0.01 mol), EEDA (0.01 mol) and ethanol (40 mL) were mixed and stirred at room temperature for 48 h and then refluxed for an additional 24 h. The concentrated solution was then precipitated in n-hexane to produce a viscous liquid followed by drying in a vacuum oven at 45 °C for 24 h. The yield of the light yellow liquid WHPA was approximately 93%.

### Synthesis of oxidized multiwalled carbon nanotubes (OMCNT)

OMCNT were prepared according to the literature with a minor modification^32^. Multiwalled carbon nanotubes (MCNT, 0.5 g) were processed in a mixture of concentrated sulfuric acid and nitric acid (3:1 v/v) under ultrasonication for 3 h at 40 °C. After that the mixture was cooled to room temperature and then diluted to 500 mL with distilled water. Subsequently, the multiwalled carbon nanotubes were filtered and rinsed with distilled water until the pH turned nearly neutral. Finally, the obtained black solid was dried under vacuum at 60 °C.

### Synthesis of WHPA functionalized OMCNT (WHPA-OMCNT)

WHPA-OMCNT was prepared according to the following steps: OMCNT (200 mg), EDC (240 mg) and NHS (500 mg) were dispersed in 500 mL of phosphate buffer saline (pH = 7.4). After the obtained mixture was stirred at room temperature for 2 h, WHPA (400 mg) was added and the reaction was continued at room temperature for 6 h. Finally, the resulting product was centrifuged (9000 rpm for 20 min) and repeatedly washed with water (two times) and ethanol (two times) to remove the free WHPA polymers that were not anchored to the nanotubes. The final product, named WHPA-OMCNT, was dried in vacuum at 55 °C for 24 h.

### General characterization

The structure and performance of the prepared materials were characterized by several techniques. ^1^H nuclear magnetic resonance spectroscopy (^1^H NMR) was recorded in a DMX-300 MHz instrument (Bruker, Germany) using CDCl_3_ as the solvent. The FTIR spectra measurements were mounted by using a Perkin-Elmer Spectrum One FTIR spectrometer (Perkin-Elmer, United States) in KBr pellet at room temperature in a spectral range of 4000–500 cm^−1^. TGA was performed on a Diamond High Temperature Type TG/DTA thermoanalyzer (Perkin-Elmer, United States) under nitrogen atmosphere from room temperature to 800 °C with a heating rate of 10 °C min^−1^. For zeta potential analysis, 5 mg of WHPA-OMCNT powder was dispersed in 10 mL of ultrapure water with various pH values. The obtained solution samples were measured with a JS94H (Shanghai, China). SEM images were recorded using a FEI QUANTA FEG250 coupled with INCA Energy X-MAX-50. Transmission electron microscopy (TEM) images were obtained from a JEOL JEM-100CX II.

### Adsorption experiments

Batch adsorption experiments were performed by taking MB, MG and MV as probes to assess the adsorption performance of WHPA-OMCNT. Water samples were prepared by dissolving known amounts of organic dyes in ultrapure water. The prepared adsorbent (WHPA-OMCNT) was placed in a beaker containing 10 mL of MB, MG or MV aqueous solution and then shaken on a temperature-controlled shaker. The dosage effect was tested in the 2–10 mg range for both MB and MV, and in the 1–6 mg range for MG (*C*_0_ = 40 mg L^−1^, contact time was 3 h, temperature was 298 K). The effect of pH was studied in the range of 2.1–9.0 for MB, 2.9–9.8 for MG and 2.1–8.1 for MV. The effect of the contact time was determined from 0.1–13.3 min for MB (*C*_0_ = 40 mg L^−1^, dosage was 5 mg, pH = 6, temperature was 298 K), 2–150 min for MG (*C*_0_ = 40 mg L^−1^, dosage was 5 mg, pH = 6, temperature was 298 K) and 2–180 min for MV (*C*_0_ = 40 mg L^−1^, dosage was 4 mg, pH = 6, temperature was 298 K). The adsorption equilibrium isotherms were determined with an initial concentration range of 40–800 mg L^−1^ for MB, MG and MV. The adsorption thermodynamics were studied at temperatures ranging from 298 to 318 K with varying initial concentrations.

At the end of the adsorption, the mixed solution was centrifuged (12000 rpm for 20 min), and the supernatant was collected to determine the residual concentrations of pollutants. The removal efficiency and the amount of pollutants adsorbed onto WHPA-OMCNT were calculated using the following equations:

1 $${\rm{Removal}}\,{\rm{efficiency}}\,( \% )\,=\,\frac{{C}_{{0}}-{C}_{e}}{{C}_{{0}}}\times 100 \% $$

2 $${{q}}_{{t}}\,=\,\frac{({C}_{{0}}-{C}_{{t}}){\rm{V}}}{{m}}$$

where *C*_*0*_ and *C*_*e*_ (mg L^−1^) are the initial and equilibrium concentrations of the pollutant, respectively. *C*_*t*_ (mg L^−1^) is the concentration of adsorbate in the aqueous solution at time *t* (min). *q*_*t*_ (mg g^−1^) is the amount of adsorbate adsorbed per unit mass of the adsorbent at time *t*. *V* (L) is the volume of the adsorbed solution, and *m* (g) is the mass of the adsorbent.

### Regeneration of the adsorbent

In the desorption experiments, an ethanol solution (25 mL) was used as the desorption agent to regenerate the adsorbents from the WHPA-OMCNT loaded with organic dyes samples. After shaking for 180 min at 298 K, the samples that separated from the solution were washed three times with ultrapure water and subjected to the next adsorption-desorption process to recycle. The adsorption-desorption cycle was successively conducted five times for each test.

### Replication of batch experiment

Each batch adsorption experiment was conducted twice and the data shown are the average values. The individual values were generally within 5%.

## Results and Discussion

### General characterization

**Figure 1:**
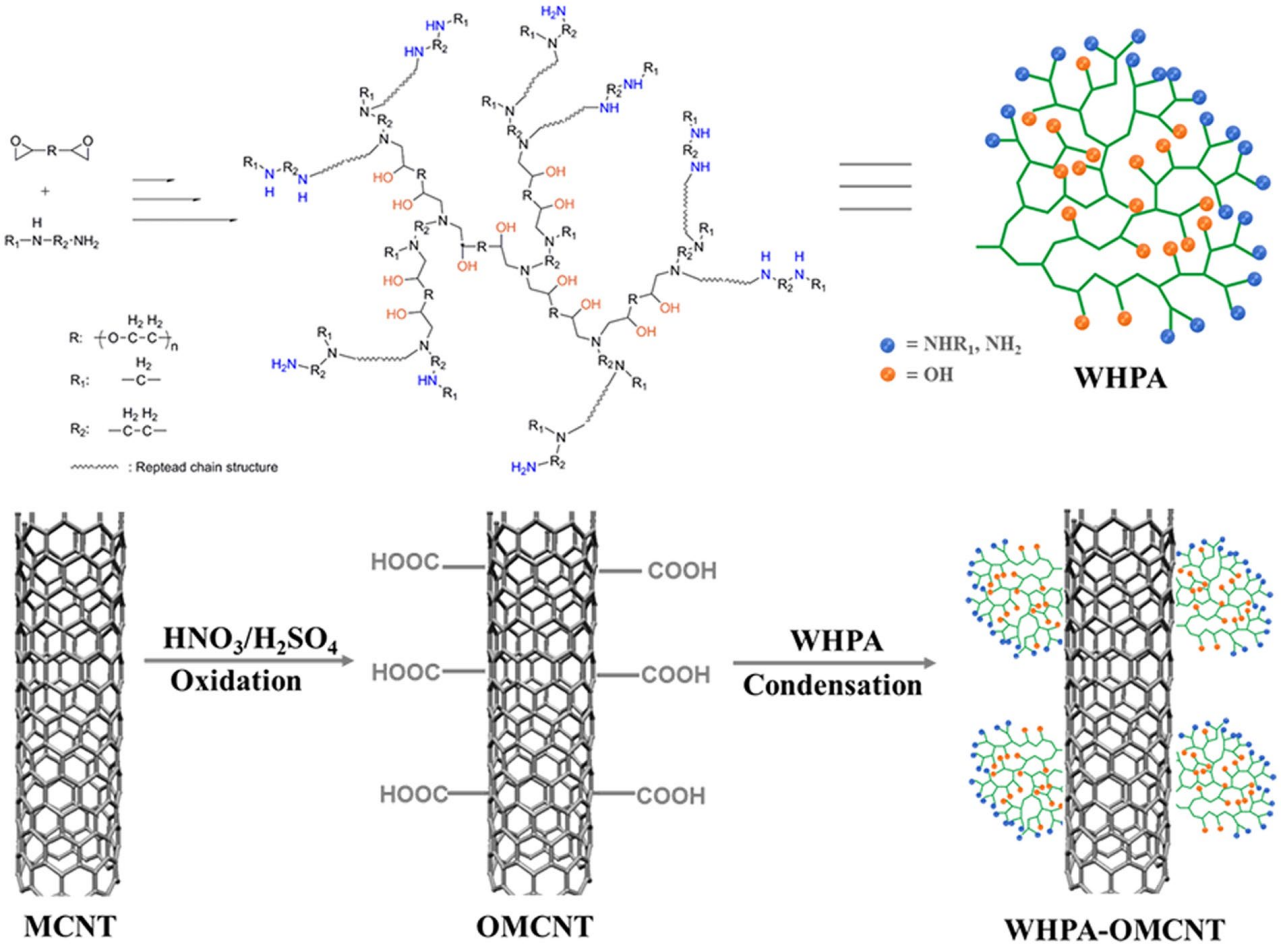
The preparation process of WHPA-OMCNT nanoadsorbent.

**Figure 2:**
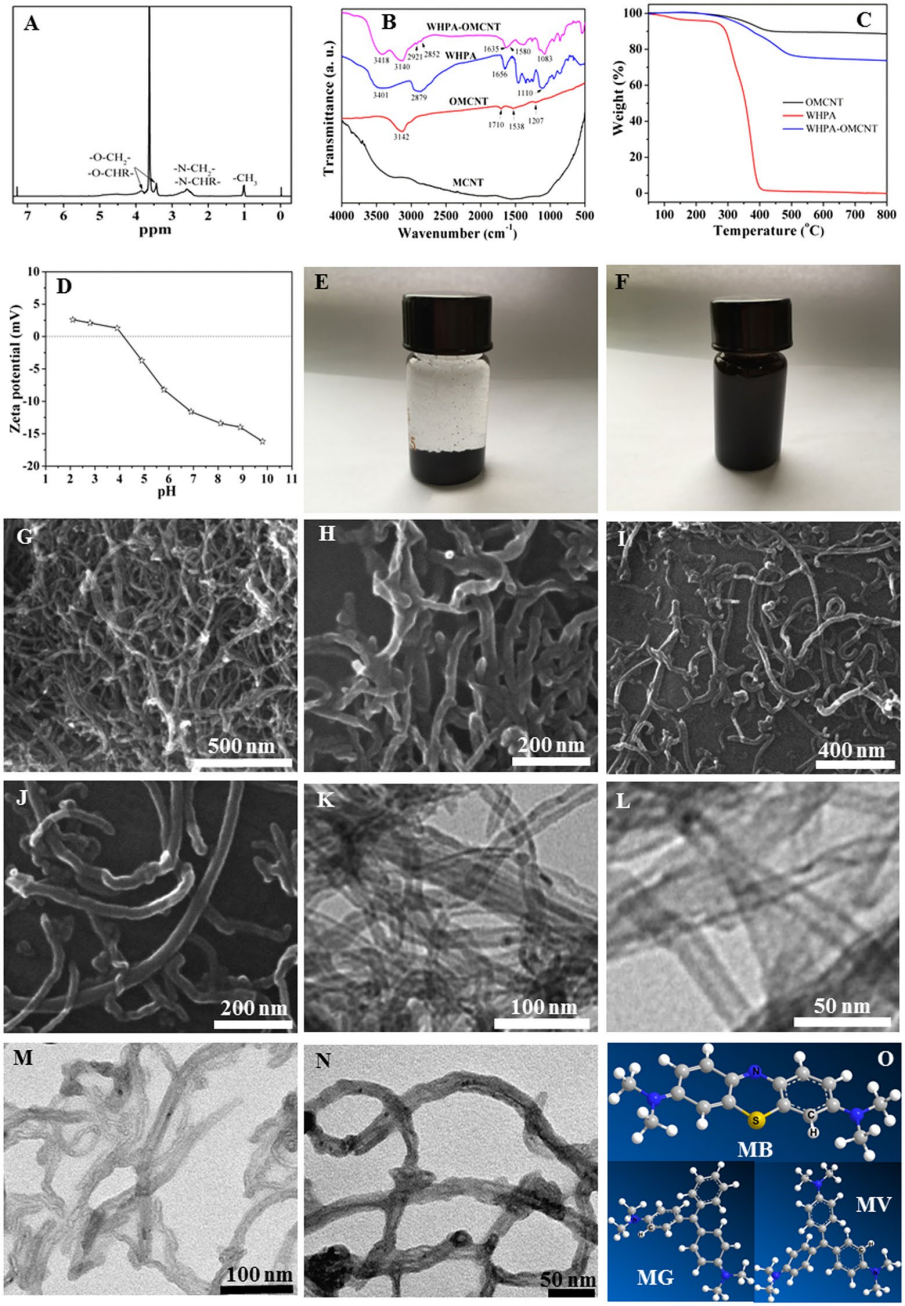
Characterization of as-prepared adsorbent: ^1^H NMR spectrum of WHPA in CDCl_3_ (**A**); FTIR spectra (**B**) and TGA analysis (**C**) of OMCNT, WHPA and WHPA-OMCNT; Zeta potential of WHPA-OMCNT (**D**); photographs for the dispersion status of OMCNT (**E**) and WHPA-OMCNT (**F**) in water settled for 3 h and two months, respectively; SEM images of OMCNT (**G,H**) and WHPA-OMCNT (**I,J**); TEM images of OMCNT (**K,L**) and WHPA-OMCNT (**M,N**); 3D models of MB, MG and MV molecules (**O**).

Figure 1 illustrates the preparation process of WHPA-OMCNT nanoadsorbent. The characterization results of the synthesized materials were shown in Fig. 2. WHPA can be easily obtained through a one-pot reaction and its molecular structure was determined by the ^1^H NMR spectrum (Fig. 2A). The peaks of -CH_3_ appeared at 0.9–1.1 ppm. In addition, the characteristic peaks at 2.2–3.1 ppm and 3.2–4.2 ppm corresponded to the signal from -CH_2_- and -CH- connected to nitrogen and oxygen atoms, respectively. These results indicated the successful synthesis of WHPA according to the literature^31^.

Figure 2B showed the FTIR spectra of origin MCNT, OMCNT, WHPA and WHPA-OMCNT. The original MCNT spectrum almost had no apparent absorption peaks. In the OMCNT spectrum, the peak at 3142 cm^−1^ could be assigned to an O-H stretch vibration. The peaks at 1710 and 1538 cm^−1^ were associated with the carboxylic acid and carboxylate anion stretch mode, and the adsorption peak at 1207 cm^−1^ corresponded to a C-OH bond^33^. These results indicated the presence of -COOH groups in OMCNT.

In the WHPA spectrum, the broad adsorption bands located at 3401 cm^−1^ were due to the stretching vibration of O-H and N-H. The adsorption band located at 2879 cm^−1^ corresponded to the stretching of the -CH_2_- groups. The peak at 1656 cm^−1^ corresponded to an N-H bending vibration of -NH_2_^34^. The characteristic peak of the aliphatic C-O ether bond appeared at 1110 cm^−1^.

The existence of abundant hydroxyl and amine groups, as well as ether bonds in WHPA, have two advantages: First, these functional groups endow WHPA with good water solubility, which is beneficial for improving the water dispersity of OMCNT. Second, the abundant hydroxyl and amine groups can interact with organic dyes. Therefore, grafting of WHPA onto OMCNT surface is expected to result in high removal efficiency of organic dyes. The covalent functionalization of OMCNT with WHPA through condensation reactions between the carboxylic acid groups of OMCNT and the amine groups of WHPA was characterized by FTIR spectroscopy. In the WHPA-OMCNT spectrum, the peak appearing at 1580 cm^−1^ corresponded to the amide (-CO-NH-), which implied the occurrence of a condensation reaction between the carboxylic acid groups of OMCNT and the amine groups of WHPA. The peak located at 1635 cm^−1^ was due to vibration of -NH_2_ groups. In addition, two new bands appeared at 2921 cm^−1^ and 2852 cm^−1^ due to -CH_2_- stretching vibrations of WHPA, which also indicated the successful grafting of WHPA onto the OMCNT surface.

In order to confirm the presence of WHPA component in WHPA-OMCNT, TGA was used to investigate the thermal stability of OMCNT, WHPA and WHPA-OMCNT. As seen from Fig. 2C, the weight loss of OMCNT starting below 200 °C was due to the volatilization of adsorbed water. A relatively large weight loss observed between 200 °C and 350 °C was attributed to elimination of oxygen-containing functional groups present on the surface of the oxidized MCNT, which was probably due to dehydration and decarboxylation^35^. In contrast, WHPA exhibited a remarkable weight loss stage from 255 °C to 450 °C, which was ascribed to degradation of the side-chains and backbone. For WHPA-OMCNT, the shape of the weight loss curve was similar to that of OMCNT. However, the onset degradation temperature (10% weight loss) of WHPA-OMCNT (377 °C) and the residue at 800 °C (73.8%) are located between those of neat OMCNT (445 °C, 88.6%) and WHPA (293 °C, 0.1%), indicating that WHPA has been successfully grafted onto the OMCNT surfaces. In addition, the WHPA-OMCNT nanocomposite adsorbent exhibited good thermal stability, which is beneficial for the application of the adsorbent.

The electronic charges on the surface of adsorbents in aqueous solutions can be analyzed by zeta potentials^36^. As shown in Fig. 2D, the point of zero charge (pHzpc) of WHPA-OMCNT was 3.8. This result indicated that when the pH was higher than 3.8, the surface of WHPA-OMCNT was negatively charged due to the deprotonation of the hydroxyl groups. Since electrostatic interactions usually dominated the adsorption process of cationic dyes, WHPA-OMCNT is expected to exhibit increased adsorption capacities in more alkaline conditions.

Due to the introduction of water-soluble HPA, WHPA-OMCNT was easily dispersed in water using ultrasonication. Figure 2E and F showed the dispersion state of OMCNT and WHPA-OMCNT in water (2 mg mL^−1^) at room temperature after settling for different periods of time. WHPA-OMCNT can be uniformly dispersed in water to form homogenous and stable solutions, and the dispersions were allowed to settle for at least two months without obvious precipitants (Fig. 2F). However, OMCNT cannot be well dispersed and precipitates formed in 3 h after ultrasonication (Fig. 2E). Therefore, the improved water solubility of WHPA-OMCNT further supports the successful functionalization of OMCNT. In addition, the well-dispersed WHPA-OMCNT is in close contact with the contaminants, which would be beneficial for high-efficient water treatment. In addition, WHPA-OMCNT water dispersion samples were centrifuged at 4000, 6000, 8000, 10000 and 12000 rpm for 20 min, respectively. The obtained photos were shown in Supplementary Fig. S1. It can be seen that due to the good water dispersion performance, WHPA-OMCNT can be completely separated from water only at very high rotation speed (12000 rpm). Therefore, 12000 rpm for 20 min were set as the parameters of centrifugation process during the following experiment.

The SEM and TEM images were used to study the morphology of OMCNT and WHPA-OMCNT. Representative images shown in Fig. 2G to Fig. 2N clearly revealed that both OMCNT and WHPA-OMCNT were tubular-like in form with an average length of hundreds of nanometers. In addition, OMCNT showed a smooth surface and aggregation state (Fig. 2G,H,K and L). However, after functionalization with water-soluble HPA, WHPA-OMCNT nanocomposite with a thin organic layer on the surface are observed, and the dispersion property of WHPA-OMCNT became better (Fig. 2I,J,M and N).

### Comparison test of different adsorbents

Different component adsorbents usually show different adsorption performances. For the sake of comparison, MCNT, OMCNT and WHPA-OMCNT were separately used to remove MB. As shown in Supplementary Fig. S2, OMCNT showed a higher adsorption capacity for MB compared with that of pristine MCNT because oxidation treatment endowed OMCNT more oxygen-containing groups that can interact with MB. In addition, WHPA-OMCNT showed a higher adsorption capacity for MB than for MG or MV. This can be explained as follows: the functionalization of OMCNT with water-soluble HPA endowed WHPA-OMCNT with better dispersity and more hydroxyl and amine groups, which increased the amounts of active sites contacted with the contaminants. It can be concluded that WHPA-OMCNT showed better removal performance for organic dyes compared with that of OMCNT and MCNT.

### Effect of dosage on the removal efficiency

**Figure 3:**
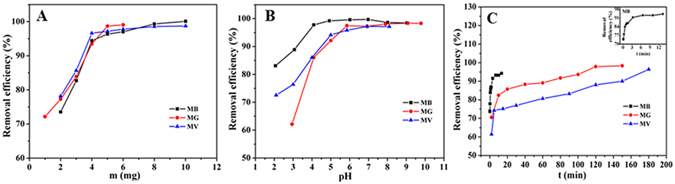
Effect of dosage (**A**), pH (**B**) and contact time (**C**) on adsorption behavior of MB, MG and MV (*C*_0_ = 40 mg L^−1^, *V* = 10 mL, temperature was 298 K).

The effect of the adsorbent dosage on the removal efficiency and adsorption capacity was investigated by adding various amounts of the WHPA-OMCNT nanocomposite to MB, MG and MV solutions followed by shaking at room temperature for 3 h (Fig. 3A). The removal efficiency of the three contaminants increased as the adsorbent dosage increased, which was due to more adsorption sites being available at higher adsorbent dosages. However, when the adsorption process reaches a saturated state, no more contaminants can be adsorbed onto the adsorbent even if the dosage of the adsorbent is increased. As indicated by the results, the removal efficiency reached equilibrium at 96.4% and 98.7% for MB and MG, respectively, corresponding to 5 mg WHPA-OMCNT dosage, 96.6% for MV at 5 mg WHPA-OMCNT dosage. Considering the removal efficiency and practicality, the optimal adsorbent dosage was maintained at 5 mg for MB and MG, and 4 mg for MV in all subsequent experiments.

### Effect of pH on the removal efficiency

Because pH has been reported as a key condition that affects the adsorption performance of organic dyes from aqueous solution^37^, a series of batch equilibrium tests were carried out to confirm the effect of pH on the adsorption of dyes by WHPA-OMCNT over a wide range of pH values. The images of wastewater before and after the removal of MB, MG and MV by the adsorbent with different pH were shown in Supplementary Fig. S3. Figure 3B shows the uptake of MB, MG and MV onto WHPA-OMCNT as a function of the corresponding solution pH. According to the obtained zeta potential results, as the pH values increased, the surface charge of WHPA-OMCNT became more negative, and the adsorption capacities of MB, MG and MV substantially increased due to electrostatic attractions between the oppositely charged ions. When the solution pH reached 6.0, the removal efficiency of MB, MG and MV was 99.6%, 97.5% and 95.9%, respectively. Subsequent increase of solution pH did not obviously increase the adsorption capacities. In addition, the pH value of the original MB, MG and MV solution was measured to be approximately 6.0. Based on the removal efficiency and simple operation, subsequent experiments were carried out with the original MB, MG and MV solution. In addition, the sorbent WHPA-OMCNT can both effectively remove the pollutants in acidic and basic solution. Therefore, we concluded that the prepared sorbent WHPA-OMCNT could be suitable for waste-water with a wide pH range.

### Effect of contact time on the removal efficiency

The contact time between the adsorbent and adsorbate is an important parameter for evaluating the adsorption properties of adsorbents. The images of wastewater before and after the removal of MB, MG and MV by the adsorbent with different time were shown in Supplementary Fig. S4. Figure 3C shows the influence of the contact time on the removal efficiency and adsorption capacities of MB, MG and MV, respectively. In general, absorption is a time-consuming process. An increase in the contact time is advantageous for sufficient interactions between the pollutants and the adsorption sites of WHPA-OMCNT. For MG and MV adsorption, the removal efficiency increased sharply within 10 min and reached equilibrium in 120 min. However, for MB adsorption, the removal efficiency increased sharply within 3 min and reached equilibrium in 10 min. In the initial stage of the adsorption process, the adsorption sites on WHPA-OMCNT for the pollutants were sufficient. As time progressed, increasing numbers of adsorption sites were occupied, and the adsorption capacity was eventually saturated. In addition, compared with that of MG and MV, the adsorption for MB reached equilibrium in a shorter time. This could be explained as follow: as illustrated in Fig. 2O, lower molecular weight and a more regular conjugate planar structure made MB molecules show smaller steric effect and better mobility, which promoted faster adsorption of MB by WHPA-OMCNT. Therefore, 10 min and 120 min were selected as the optimum contact time for MB and MG/MV removal, respectively.

### Adsorption kinetics

The kinetics of the contaminant adsorbing onto WHPA-OMCNT was described by four adsorption equations^38–41^. Each model is expressed as follows:

- Pseudo-first order model:

3 $$\mathrm{lg}({q}_{{\rm{e}}}-{q}_{{\rm{t}}})=\,\mathrm{lg}\,{q}_{{\rm{e}}}-{k}_{1}t$$

- Pseudo-second order model:

4 $$\frac{t}{{q}_{{\rm{t}}}}=\frac{1}{{k}_{2}{q}_{{\rm{e}}}^{{\rm{2}}}}+\frac{t}{{q}_{{\rm{e}}}}$$

- Intraparticle diffusion model:

5 $${q}_{{\rm{t}}}={k}_{{\rm{dif}}}{t}^{{\rm{1}}/{\rm{2}}}+{\rm{C}}$$

- Bangham model:

6 $$\mathrm{ln}\,{q}_{{\rm{t}}}=\,\mathrm{ln}\,{k}_{{\rm{b}}}+(\frac{1}{m})\mathrm{ln}\,t$$

where *q*_e_ and *q*_t_ (mg g^−1^) are the amount of pollutants adsorbed onto the adsorbent at equilibrium and at time *t* (min), respectively. *k*_1_ and *k*_2_ (mg min g^−1^) are the pseudo-first order and pseudo-second order rate constant, respectively. *h* (*h* = *k*_2_ *q*_e_^2^_)_ is the initial sorption rate (mg g^−1^ min^−1^), which indicate the movement rate of each dye molecule. *k*_dif_ (mg g^−1^ min^−1/2^) is the intraparticle diffusion rate constant. *m* and *k*_b_ are the related constants of the Bangham model.

**Figure 4:**
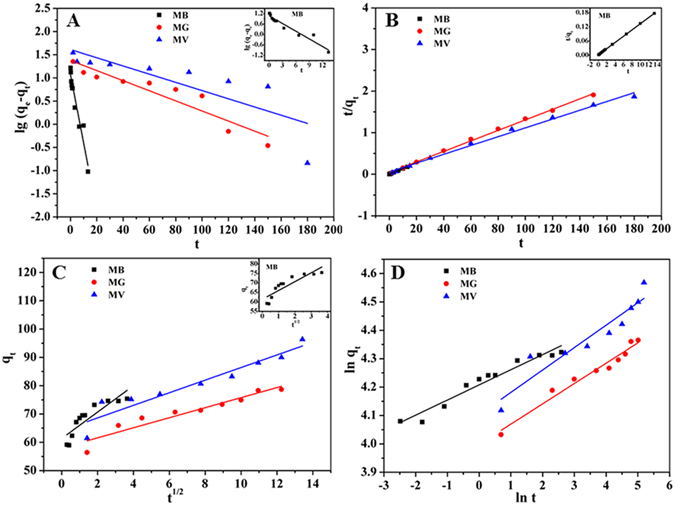
Pseudo-first-order kinetics (**A**), Pseudo-second-order kinetics (**B**), Intraparticle diffusion kinetics (**C**) and Bangham kinetics (**D**) for adsorption of MB and MG (*m* = 5 mg, *C*_0_ = 40 mg L^−1^, *V* = 10 mL, pH = 6, temperature was 298 K), MV (*m* = 4 mg, *C*_0_ = 40 mg L^−1^, *V* = 10 mL, pH = 6, temperature was 298 K).

Figure 4 shows the linear fitting results of the kinetic data, and the relevant calculated results are listed in Supplementary Table S1. All of the obtained experimental data fitted better with the pseudo-second order kinetic model than with the three other models (MB: R^2^ = 0.9999, MG: R^2^ = 0.9976, MV: R^2^ = 0.9934), which indicated that the adsorption rate was primarily controlled by chemisorption. In addition, the calculated *q*_e_ from the pseudo-second order kinetic model (75.6 mg g^−1^ for MB, 79.1 mg g^−1^ for MG and 94.4 mg g^−1^ for MV) are consistent with the experimental data (75.4 mg g^−1^ for MB, 78.7 mg g^−1^ for MG and 96.4 mg g^−1^ for MV).

In addition, the dependence of the movement of each dye molecule on the temperature in the adsorption process was further investigated through Pseudo second-order kinetic model study. The adsorption experiments of MB, MG and MV onto WHPA-OMCNT for different time at different temperature were conducted and the results were shown in Supplementary Fig. S5 and Supplementary Table S2. It can be seen that values of pseudo second-order rate constant (*k*) and initial adsorption rate (*h*) increased with increasing temperature. Higher temperature favors the adsorption process by increasing sorption rate^42^.

### Adsorption isotherms

In order to provide insight into the adsorption behavior of an adsorbent, four isotherm equations were selected to model the adsorption isotherm data including the Henry^43^, Langmuir^44^, Freundlich^45^ and Dubinin-Radushkevich (D-R) equations^46^, which can be expressed as follows:

- Henry equation:

7 $${q}_{{\rm{e}}}={K}_{{\rm{H}}}{C}_{{\rm{e}}}$$

- Langmuir equation:

8 $$\frac{{C}_{{\rm{e}}}}{{q}_{{\rm{e}}}}\,=\,\frac{1}{b{q}_{{\rm{m}}}}\,+\,\frac{{C}_{{\rm{e}}}}{{q}_{{\rm{m}}}}$$

- Freundlich equation:

9 $$\mathrm{ln}\,{q}_{{\rm{e}}}=\,\mathrm{ln}\,{K}_{{\rm{F}}}\,+\,\frac{1}{n}\,\mathrm{ln}\,{C}_{{\rm{e}}}$$

- Dubinin-Radushkevich equation:

10 $$\mathrm{ln}\,\,{q}_{e}=\,\mathrm{ln}\,{q}_{m}-\beta {\varepsilon }^{2}$$

where *q*_m_ (mg g^−1^) is the maximum adsorption capacity. *K*_H_ and *K*_F_ are the constants related to the adsorption capacity and intensity, respectively. *b* (L mg^−1^) is the Langmuir constant related to the affinity of the binding site. A smaller 1/*n* value indicates a more heterogeneous surface. However, a value closer to or equal to one indicates the adsorbent has relatively more homogeneous binding sites. $\frac{{RT}}{{{b}}_{{T}}}$ is related to the adsorption heat. *A*_*T*_ is the equilibrium constant corresponding to the maximum binding energy. *β* (mol^2^ kJ^−2^) is the average free energy generated per gram adsorbent. *ε* is the Polanyi potential energy.

**Figure 5:**
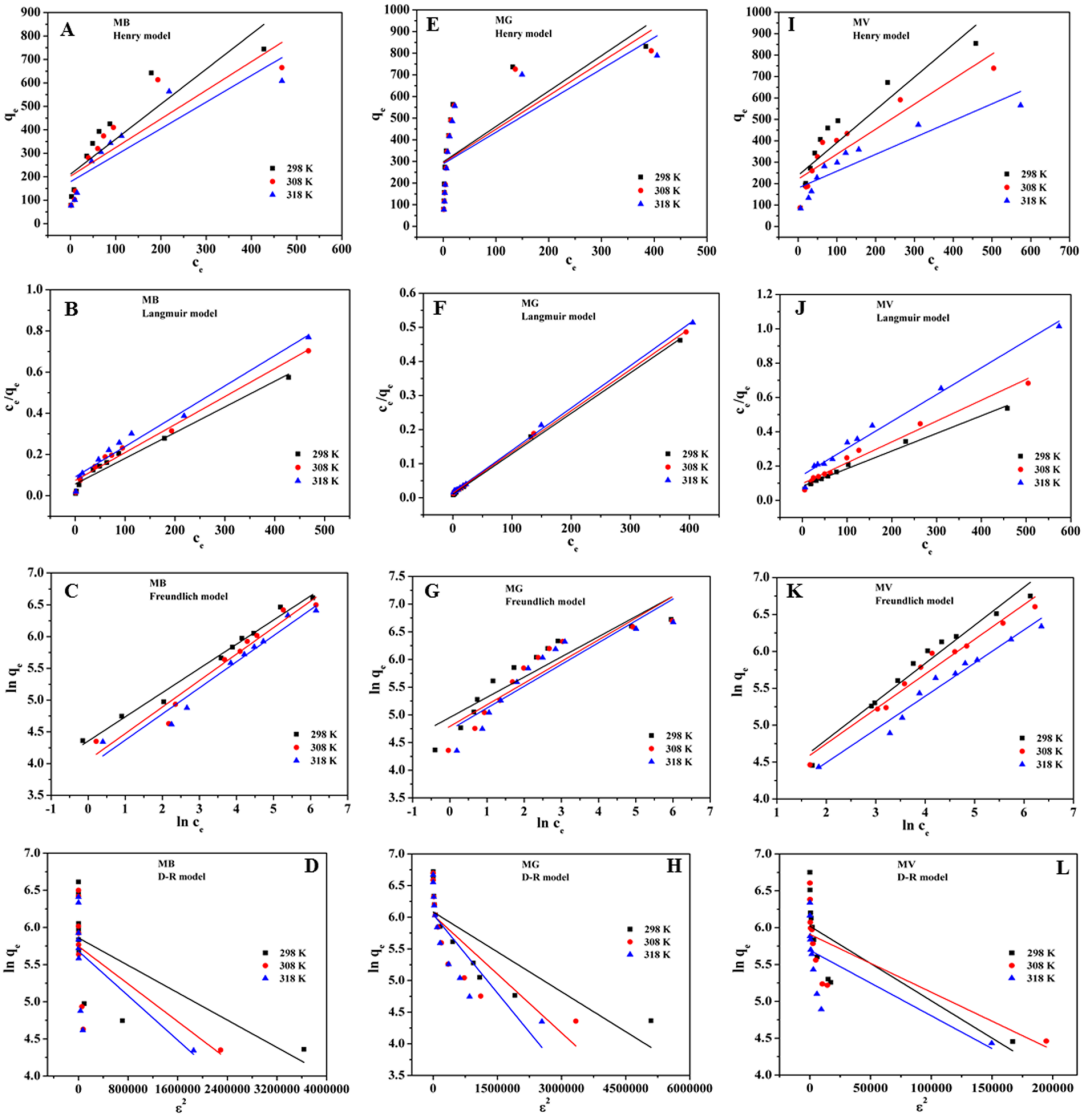
Henry (**A**), Langmuir (**B**), Freundlich (**C**) and D-R (**D**) adsorption isotherm fit of MB (*m* = 5 mg, *C*_0_ = 40–800 mg L^−1^, *V* = 10 mL, pH = 6, contact time was 10 min, temperature was 298 K, 308 K and 318 K, respectively); Henry (**E**), Langmuir (**F**), Freundlich (**G**) and D-R (**H**) adsorption isotherm fit of MG (*m* = 5 mg, *C*_0_ = 40–800 mg L^−1^, *V* = 10 mL, pH = 6, contact time was 120 min, temperature was 298 K, 308 K and 318 K, respectively) and Henry (**I**), Langmuir (**J**), Freundlich (**K**) and D-R (**L**) adsorption isotherm fit of MV (*m* = 4 mg, *C*_0_ = 40–800 mg L^−1^, *V* = 10 mL, pH = 6, contact time was 120 min, temperature was 298 K, 308 K and 318 K, respectively).

The fitted results of all isotherm models are presented in Fig. 5, and the calculated parameters are listed in Supplementary Table S3. According to the correlation coefficients (R^2^), the Langmuir model is more suitable than the three other models for describing the adsorption of MB (R^2^ = 0.9644, R^2^ = 0.9766, R^2^ = 0.9686), MG (R^2^ = 0.9989, R^2^ = 0.9994, R^2^ = 0.9991) and MV (R^2^ = 0.9888, R^2^ = 0.9785, R^2^ = 0.9805) at 298 K, 308 K and 318 K, respectively. These results suggested that the surface of WHPA-OMCNT was covered by monolayer pollutant, and the reaction interface between MB/MG/MV and WHPA-OMCNT was nonhomogeneous. The maximum adsorption amount calculated from the Langmuir model was 800.0 mg g^−1^ for MB, 840.3 mg g^−1^ for MG and 970.9 mg g^−1^ for MV at 298 K. The 1/*n* value is an indicator of the favorite state of the absorption process. The smaller 1/*n* value indicated that MB, MG or MV was easily adsorbed onto the heterogeneous surface of the WHPA-OMCNT composite.

### Thermodynamic parameters

Thermodynamic studies provide in-depth information on inherent energetic changes during the adsorption process. In this work, the effects of the temperature on the adsorption were investigated, and the thermodynamic behavior was evaluated using the following equations:

11 $${\rm{\Delta }}{G}=-{RT}\,\mathrm{ln}\,{{K}}_{{d}}$$

12 $$\mathrm{ln}\,{{K}}_{{d}}=\frac{{\rm{\Delta }}{S}}{{R}}-\frac{{\rm{\Delta }}{H}}{{RT}}$$

where *R* (8.314 J mol^−1^ K^−1^) is the gas constant, T (K) is the absolute temperature and *K*_d_ is the thermodynamic equilibrium constant. Δ*S* is the entropy change, Δ*H* is the enthalpy change, and Δ*G* is the Gibbs free energy change of a given process (kJ mol^−1^). The Δ*H*, Δ*S* and Δ*G* results are shown in Supplementary Table S4. The negative values of Δ*H* demonstrated the exothermic nature of the adsorption processes. The negative values of Δ*S* indicated that MB, MG or MV molecules were orderly adsorbed onto the WHPA-OMCNT. The negative values of Δ*G* suggested the spontaneous nature of the adsorption processes at the three temperatures.

### Adsorption mechanism

**Figure 6:**
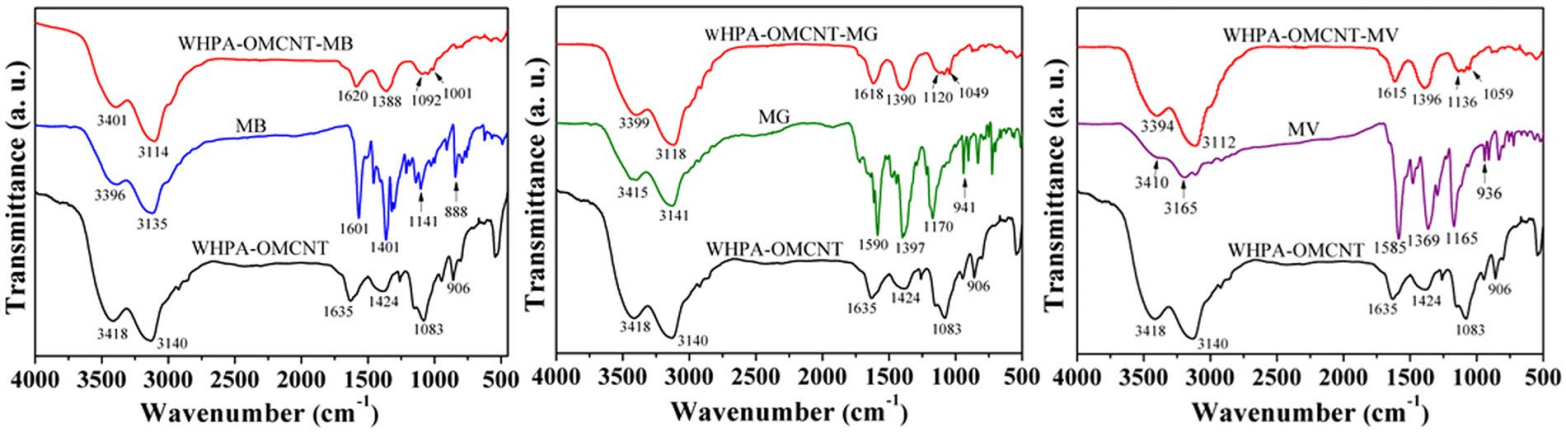
FTIR spectra of WHPA-OMCNT before and after loading MB (WHPA-OMCNT-MB), MG (WHPA-OMCNT-MG) and MV (WHPA-OMCNT-MV).

**Figure 7:**
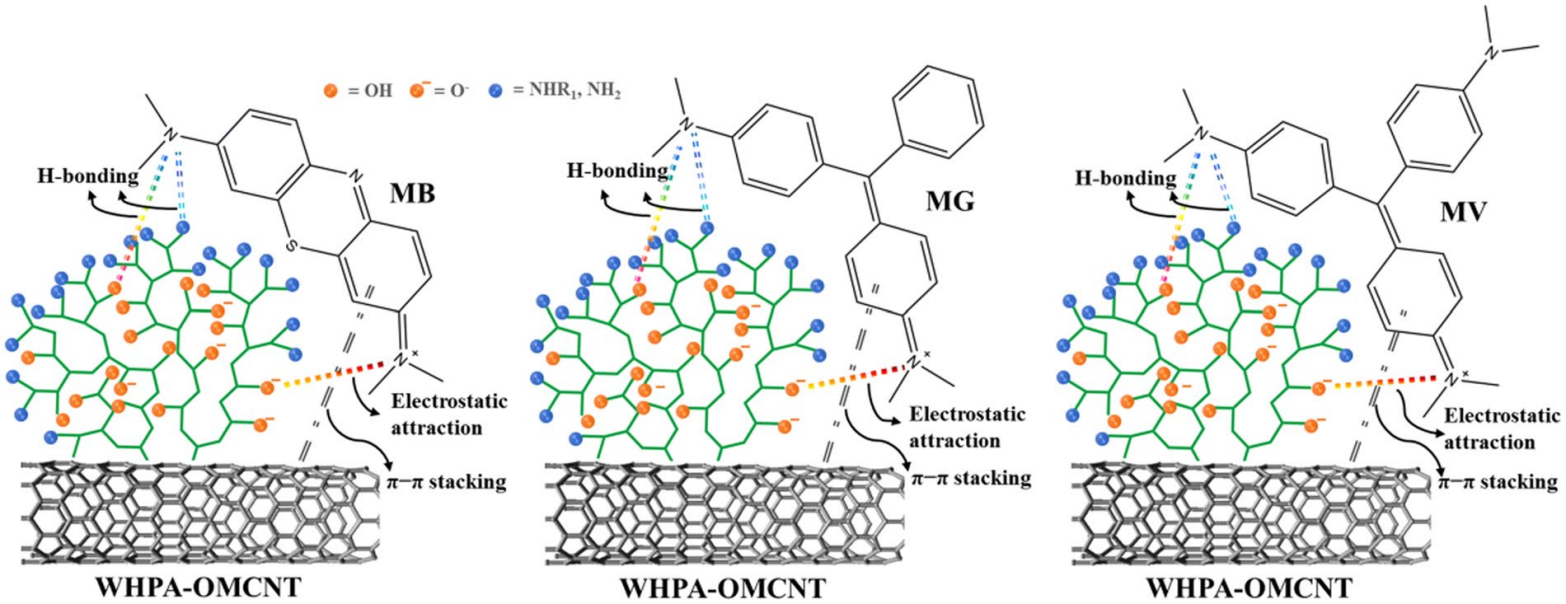
Proposed adsorption mechanism of MB, MG and MV adsorption onto WHPA-OMCNT.

In order to illustrate the adsorption mechanism, FTIR spectra of WHPA-OMCNT nanocomposites with adsorbed MB, MG and MV (referred to as WHPA-OMCNT-MB, WHPA-OMCNT-MG and WHPA-OMCNT-MV, respectively) were recorded to study the possible interaction sites between adsorbent and adsorbate molecule (Fig. 6). The peak related to the vibration of the aromatic ring located at 1601 cm^−1^ for MB, 1590 cm^−1^ for MG and 1585 cm^−1^ for MV; the C-N stretching vibration located at 1401 cm^−1^ for MB, 1397 cm^−1^ for MG and 1369 cm^−1^ for MV; the C-H in plane and out of plane bending vibrations located at 1141 cm^−1^ and 888 cm^−1^ for MB, 1170 cm^−1^ and 941 cm^−1^ for MG and 1165 cm^−1^ and 936 cm^−1^ for MV^47^. In addition, characteristic peak shifts were observed in the spectra after adsorption. For MB, MG and MV removal, the peaks at 3418 cm^−1^ and 3140 cm^−1^ corresponding to the -OH and -NH_2_ stretching vibration shifted to 3401 cm^−1^ and 3114 cm^−1^ (for MB), 3399 cm^−1^ and 3118 cm^−1^ (for MG) and 3394 cm^−1^ and 3112 cm^−1^ (for MV), indicating the electrostatic attractions as well as H-bonding between the active sites of cationic MB, MG, and MV dyes and the hydroxyls groups and amine groups of WHPA-OMCNT nanocomposites^22, 48–51^. Moreover, the adsorption bands 1635 cm^−1^ corresponding to the N-H bending vibration shifted to 1620 cm^−1^ (for MB), 1618 cm^−1^ (for MG) and 1615 cm^−1^ (for MV), suggesting that H-bonding may occur between the dye molecules and WHPA-OMCNT^22, 48^. In addition, it is believed that the π-π stacking interactions between the hexagonal skeleton of WHPA-OMCNT and aromatic rings of organic dyes can also be beneficial to the adsorption of the three dyes^15, 49, 51–53^. A possible mechanism has been proposed in Fig. 7.

### Performance evaluation

The effect of common metal ions, such as K^+^, Na^+^, Ca^2+^ and Mg^2+^, on the adsorption of dyes was investigated using KNO_3_, NaNO_3_, Ca(NO_3_)_2_ and Mg(NO_3_)_2_ as the ionic medium. In the present study, different concentrations of K^+^, Na^+^, Ca^2+^ and Mg^2+^ were utilized in solutions with otherwise constant parameters (adsorbent dosage 5 mg, volume 10 mL, pH 6, contact time 10 min, temperature 298 K, dye concentration 40 mg L^−1^ for MB; adsorbent dosage 5 mg, volume 10 mL, pH 6, contact time 120 min, temperature 298 K, dye concentration 40 mg L^−1^ for MG and adsorbent dosage 4 mg, volume 10 mL, pH 6, contact time 120 min, temperature 298 K, dye concentration 40 mg L^−1^ for MV). Their presence may compete with MB, MG or MV for binding at the adsorption sites on the surface of WHPA-OMCNT^54^. According to the results shown in Supplementary Fig. S6, the removal efficiency of the three pollutants decreased slightly along with the increased coexisting ions, which indicated that the high-concentration coexisting ions weakly interfered with MB, MG and MV adsorption. However, the decrease is still acceptable compared to the high removal efficiency of the pollutants.

**Figure 8:**
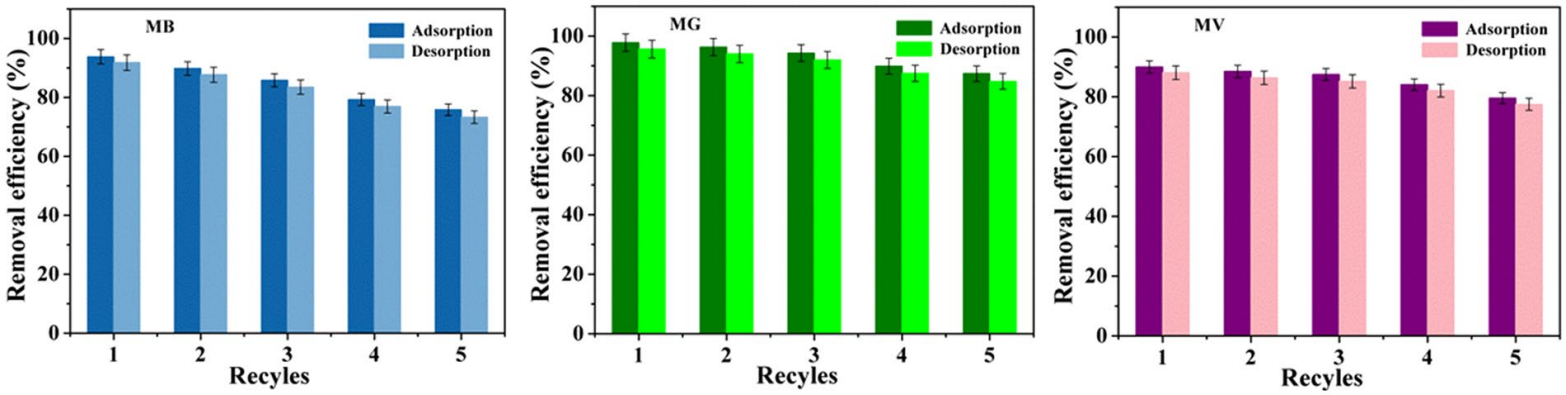
Adsorption-desorption recycles of MB (*m = *5 mg, *C*_0_ = 40 mg L^−1^, *V = *10 mL, pH = 6, contact time was 10 min, temperature was 298 K), MG (*m = *5 mg, *C*_0_ = 40 mg L^−1^, *V = *10 mL, pH = 6, contact time was 120 min, temperature was 298 K) and MV (*m = *4 mg, *C*_0 = _40 mg L^−1^, *V = *10 mL, pH = 6, contact time was 120 min, temperature was 298 K). Error bar = SD (n = 2).

The recycling and regeneration abilities of the adsorbent are important for evaluating their performance for practical applications. In this study, the inexpensive reagents, ethanol and water were used to desorb the adsorbed dyes. As a result, ethanol was observed to be an effective desorption agent to recover MB, MG and MV from the WHPA-OMCNT adsorbent (Supplementary Fig. S7). After five replicates of adsorption-desorption recycling experiments, WHPA-OMCNT still exhibited acceptable removal efficiency for all three pollutants with a gradual decrease (Fig. 8). Therefore, WHPA-OMCNT promised a great potential for easy recycling and reuse for wastewater treatment. In addition, the TEM images of WHPA-OMCNT adsorbents before and after MB dye removal, the first and the fifth recycled ones were depicted in Supplementary Fig. S8. It can been seen that after the first adsorption of MB, the diameter of the loaded adsorbent WHPA-OMCNT-MB (Supplementary Fig. S8B) was larger than that of WHPA-OMCNT (Supplementary Fig. S8A), which can be attributed to the accumulation of MB dye molecules over the adsorbent surface. After the first desorption by ethanol, the MB dye molecules on the adsorbent surface were almost desorbed (Supplementary Fig. S8C). However, after five replicates of adsorption–desorption recycling experiments, a small amount of the MB dye adsorbed on the WHPA-OMCNT surface cannot be desorbed (Supplementary Fig. S8D). So the desorption efficiency in the recycling experiments declined gradually.

In addition, the maximum adsorption capacities of WHPA-OMCNT nanocomposites for MB, MG and MV were compared with those of other reported adsorbents to illustrate the excellent adsorption performance of WHPA-OMCNT. As seen from Supplementary Table S5, the maximum adsorption capacities of WHPA-OMCNT for MB, MG and MV were higher than those of other adsorbents. This can be explained from two aspects: First, CNT has a high specific surface area, which increases the contact area between the adsorbent and adsorbate. Second, the existence of water-soluble hyperbranched polyamine endowed WHPA-OMCNT with good water dispersity and abundant hydroxyl and amine groups, which can strengthen the interaction between the adsorbent and the adsorbate. Therefore, WHPA-OMCNT can effectively improve the adsorption capacity of MB, MG and MV. Considering the convenient synthetic process and good adsorption performance, WHPA-OMCNT is a good absorbent for polluted water treatment.

## Conclusions

In this study, a simple and effective approach based on surface modification of carbon nanotubes with a water-soluble hyperbranched polyamine was developed to obtain a WHPA-OMCNT adsorbent. WHPA-OMCNT exhibited good removal properties of MB, MG and MV from aqueous solutions. The equilibrium adsorption capacity of WHPA-OMCNT was 800.0 mg g^−1^ for MB, 840.3 mg g^−1^ for MG and 970.9 mg g^−1^ for MV at 298 K. The results from the adsorption kinetics and isotherm studies indicated that the adsorption processes of these three types of pollutants fitted well with the pseudo-second order equation and Langmuir model, respectively. In addition, the thermodynamic studies indicated that the adsorption process was exothermic and spontaneous in nature. WHPA-OMCNT can be effectively regenerated by inexpensive reagent and retained considerable adsorption capacity after several adsorption-desorption cycles. Based on the high efficiency and feasibility, WHPA-OMCNT exhibited a great potential for water purification.

## Electronic supplementary material

Supplementary information

## References

[1] Miksch K (2015). R&D priorities in the field of sustainable remediation and purification of agro-industrial and municipal wastewater. New Biotechnol..

[2] Zollinger, H. Color Chemistry: Synthesis, properties and applications of organic dyes and pigments. (1987).

[3] Forgacs E, Cserhati T, Oros G (2004). Removal of synthetic dyes from wastewaters: A review. Environ. Int..

[4] Chen H, Wang XX, Li JX, Wang XK (2015). Cotton derived carbonaceous aerogels for the efficient removal of organic pollutants and heavy metal ions. J. Mater. Chem. A.

[5] Yagub MT, Sen TK, Afroze S, Ang HM (2014). Dye and its removal from aqueous solution by adsorption: a review. Adv. Colloid Interface Sci..

[6] Demirbas A (2009). Agricultural based activated carbons for the removal of dyes from aqueous solutions: A review. J. Hazard. Mater..

[7] Wang SB, Peng YL (2010). Natural zeolites as effective adsorbents in water and wastewater treatment. Chem. Eng. J..

[8] Haderlein SB, Weissmahr KW, Schwarzenbach RP (1996). Specific adsorption of nitroaromatic explosives and pesticides to clay minerals. Environ. Sci. Technol..

[9] Doğan M, Özdemir Y, Alkan M (2007). Adsorption kinetics and mechanism of cationic methyl violet and methylene blue dyes onto sepiolite. Dyes Pigm..

[10] Chen S (2014). Aqueous cationic, anionic and non-ionic multi-walled carbon nanotubes, functionalised with minimal framework damage, for biomedical application. Biomaterials.

[11] Wan Q (2015). Mussel inspired preparation of highly dispersible and biocompatible carbon nanotubes. RSC Adv..

[12] Yang SB, Han C, Wang XK, Nagatsu M (2014). Characteristics of cesium ion sorption from aqueous solution on bentonite- and carbon nanotube-based composites. J. Hazard. Mater..

[13] Ren XM, Chen CL, Nagatsu M, Wang XK (2011). Carbon nanotubes as adsorbents in environmental pollution management: A review. Chem. Eng. J..

[14] Wang XX, Chen CL, Li JK, Wang XK (2015). Ozone degradation of 1-naphthol on multiwalled carbon nanotubes/iron oxides and recycling of the adsorbent. Chem. Eng. J..

[15] Ai LH (2012). Removal of methylene blue from aqueous solution with magnetite loaded multi-wall carbon nanotube: Kinetic, isotherm and mechanism analysis. J. Hazard. Mater..

[16] Sheng GD (2016). Enhanced sequestration of selenite in water by nanoscale zero valent iron immobilization on carbon nanotubes by a combined batch, XPS and XAFS investigation. Carbon.

[17] Jalil G, Mokhtar A, Hajir B, Mohammad MN (2014). Modification of carbon nanotubes with cationic surfactant and its application for removal of direct dyes. Desalin. Water Treat.

[18] Ghaedi M, Hajati S, Zare M, Zarec M, Jaberi SYS (2015). Experimental design for simultaneous analysis of malachite green and methylene blue; derivative spectrophotometry and principal component artificial neural network. RSC Adv..

[19] Yang SB, Hu J, Chen CL, Shao DD, Wang XK (2011). Mutual effects of Pb(II) and humic acid adsorption on multiwalled carbon nanotubes/polyacrylamide composites from aqueous solutions. Environ. Sci. Technol..

[20] Shang SM, Gan L, Yuen MCW (2013). Improvement of carbon nanotubes dispersion by chitosan salt and its application in silicone rubber. Compos. Sci. Technol.

[21] Shang SM, Gan L, Yuen MCW, Jiang SX, Luo NM (2014). Carbon nanotubes based high temperature vulcanized silicone rubber nanocomposite with excellent elasticity and electrical properties. Compos. Part A-Appl. S.

[22] Ghorai S (2014). Enhanced removal of methylene blue and methyl violet dyes from aqueous solution using a nanocomposite of hydrolyzed polyacrylamide grafted xanthan gum and incorporated nanosilica. ACS Appl. Mater. Interfaces.

[23] Gao HJ, Zhao SY, Cheng XY, Wang XD, Zheng LQ (2013). Removal of anionic azo dyes from aqueous solution using magnetic polymer multi-wall carbon nanotube nanocomposite as adsorbent. Chem. Eng. J..

[24] Chatterjee S, Chatterjee T, Li SR, Woo SH (2011). Effect of the addition mode of carbon nanotubes for the production of chitosan hydrogel core-shell beads on adsorption of Congo red from aqueous solution. Bioresource Technol..

[25] Xie YL (2015). Carbon nanotube based polymer nanocomposites: biomimic preparation and organic dye adsorption applications. RSC Adv..

[26] Gao C, Yan DY (2014). Hyperbranched polymers: from synthesis to applications. Prog. Polym. Sci..

[27] Zhou L, Gao C, Xu WJ (2010). Magnetic dendritic materials for highly efficient adsorption of dyes and drugs. ACS Appl. Mater. Interfaces.

[28] Qiang TT, Luo M, Bu QQ, Wang XC (2012). Adsorption of an acid dye on hyperbranched aminated collagen fibers. Chem. Eng. J..

[29] Niu YZ (2013). Adsorption of Pb(II) from aqueous solution by silica-gel supported hyperbranched polyamidoamine dendrimers. J. Hazard. Mater..

[30] Hu, L.H. *et al*. Fabrication of magnetic water-soluble hyperbranched polyol functionalized graphene oxide for high-efficiency water remediation. *Sci. Rep*. doi:10.1038/srep28924. 10.1038/srep28924 PMC4926210 27354318

[31] Yu B, Jiang XS, Yin GL, Yin J (2014). Multistimuli-responsive hyperbranched poly(ether amine)s. J. Polym. Sci. Pol. Chem..

[32] Cui JW, Liu YQ, Hao JC (2009). Multiwalled carbon-nanotube-embedded microcapsules and their electrochemical behavior. J. Phys. Chem. C.

[33] Gong J (2014). Catalytic carbonization of polypropylene into cup-stacked carbon nanotubes with high performances in adsorption of heavy metallic ions and organic dyes. Chem. Eng. J..

[34] Liu XS (2013). Mussel-inspired polydopamine: a biocompatible and ultrastable coating for nanoparticles *in vivo*. ACS Nano.

[35] Datsyuk V (2008). Chemical oxidation of multiwalled carbon nanotubes. Carbon.

[36] Moussavi G, Barikbin B (2010). Biosorption of chromium(VI) from industrial wastewater onto pistachio hull waste biomass. Chem. Eng. J..

[37] Gui CX (2014). Sandwich like magnesium silicate/reduced graphene oxide nanocomposite for enhanced Pb^2+^ and methylene blue adsorption. ACS Appl. Mater. Interfaces.

[38] Wang H, Yu YF, Chen QW, Cheng K (2010). Carboxyl-functionalized nanoparticles with magnetic core and mesopore carbon shell as adsorbents for the removal of heavy metal ions from aqueous solution. Dalton Trans..

[39] Ho YS (2006). Review of second-order models for adsorption systems. J. Hazard. Mater..

[40] Kumar R, Barakat M, Daza Y, Woodcock H, Kuhn J (2013). EDTA functionalized silica for removal of Cu(II), Zn(II) and Ni(II) from aqueous solution. J. Colloid Interface Sci..

[41] Repo E, Warchoł JK, Bhatnagar A, Sillanp M (2011). Heavy metals adsorption by novel edta-modified chitosan-silica hybrid materials. J. Colloid Interface Sci..

[42] Celekli A, Ilgun G, Bozkurt H (2012). Sorption equilibrium, kinetic, thermodynamic, and desorption studies of Reactive Red 120 on Chara contraria. Chem. Eng. J..

[43] Larson CP, Eger EI, Severinghaus JW (1962). The solubility of halothane in blood and tissue homogenates. Anesthesiology.

[44] Langmuir I (1918). The adsorption of gases on plane surfaces of glass, mica and platinum. J. Am. Chem. Soc..

[45] Freundlich HMF (1906). Over the adsorption in solution. J. Phys. Chem..

[46] Dubinin MM, Radushkevich LV (1947). Equation of the characteristic curve of activated charcoal. Chem. Zentr..

[47] Yan YM (2005). Adsorption of methylene blue dye onto carbon nanotubes: a route to an electrochemically functional nanostructure and its layer-by-layer assembled nanocomposite. Chem. Mater..

[48] Zhao Y, Chen HL, Li J, Chen CL (2015). Hierarchical MWCNTs/Fe_3_O_4_/PANI magnetic composite as adsorbent for methyl orange removal. J. Colloid Interface Sci..

[49] Liu F, Chung SY, Oh G, Seo TS (2012). Three-dimensional graphene oxide nanostructure for fast and efficient water-soluble dye removal. ACS Appl. Mater. Interfaces.

[50] Feng M, You W, Wu ZS, Chen QD, Zhan HB (2013). Mildly alkaline preparation and methylene blue adsorption capacity of hierarchical flower-like sodium titanate. ACS Appl. Mater. Interfaces.

[51] Ma J (2012). Enhanced adsorptive removal of methyl orange and methylene blue from aqueous solution by alkali-activated multiwalled carbon nanotubes, enhanced adsorptive removal of methyl orange and methylene blue from aqueous solution by alkali-activated multiwalled carbon nanotubes. ACS Appl. Mater. Interfaces.

[52] Ren X (2015). One-pot polymer conjugation on carbon nanotubes through simultaneous π−π stacking and the Biginelli reaction. Polymer.

[53] Lin DH, Xing BS (2008). Adsorption of phenolic compounds by carbon nanotubes: role of aromaticity and substitution of hydroxyl groups. Environ. Sci. Technol..

[54] Ma YL, Xu ZR, Guo T, You P (2004). Adsorption of methylene blue on Cu(II)-exchanged montmorillonite. J. Colloid Interface Sci..

