# Supplementary material for: Facile preparation of water-soluble hyperbranched polyamine functionalized multiwalled carbon nanotubes for high-efficiency organic dye removal from aqueous solution
Source: Sci Rep. 2017 Jun 15;7:3611. doi: 10.1038/s41598-017-03490-6 (PMC5472635; doi:10.1038/s41598-017-03490-6)
Supplement: Supplementary file 1 — Supplementary information [file 41598_2017_3490_MOESM1_ESM.pdf]

Supplementary information

Facile preparation of water-soluble hyperbranched polyamine functionalized multiwalled carbon nanotubes for high-efficiency organic dye removal from aqueous solution

Lihua Hu [a], Zhongping Yang [a], Yaoguang Wang [a], Yan Li [a], Dawei Fan [a], Di Wu [b], Qin Wei [a], Bin Du [b]*

[a] Key Laboratory of Chemical Sensing & Analysis in Universities of Shandong, School of Chemistry and Chemical Engineering, University of Jinan, Jinan 250022, PR China

[b] School of Resources and Environment, University of Jinan, Jinan 250022, PR China

*Corresponding author. Tel. + 86-531-82767370; fax: + 86-531-82767370.

E-mail address: dubin61@gmail.com (Bin Du).

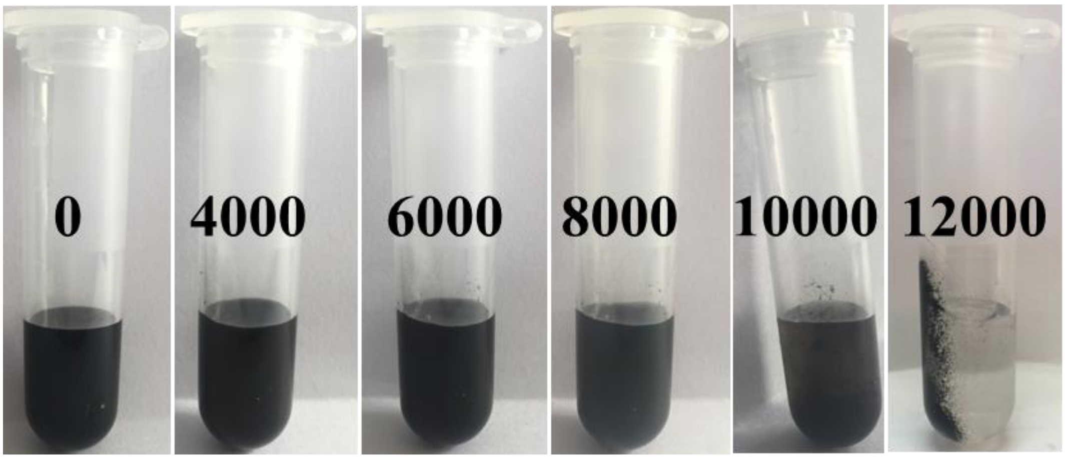

Figure S1. The photos of WHPA-OMCNT water dispersion samples centrifuged at 0, 4000, 6000, 8000, 10000 and 12000 rpm for 20 min, respectively. (C = 2 mg mL^{-1}).

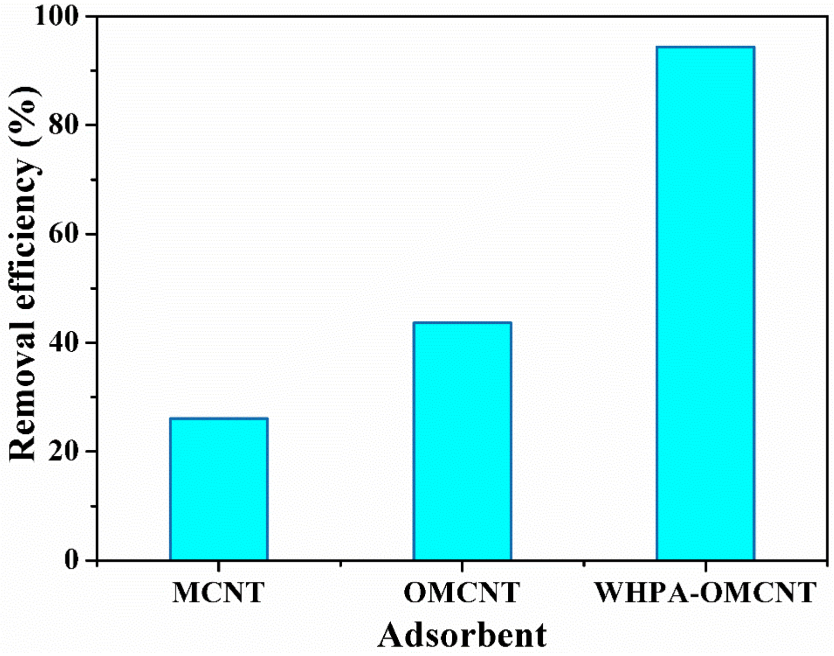

Figure S2. Removal efficiency of MB by using CNT, OMCNT and WHPA-OMCNT adsorbent, respectively. (C_0 = 40 mg L^{-1}, V = 10 mL, dosage = 4 mg, contact time was 3 h, temperature was 298 K).

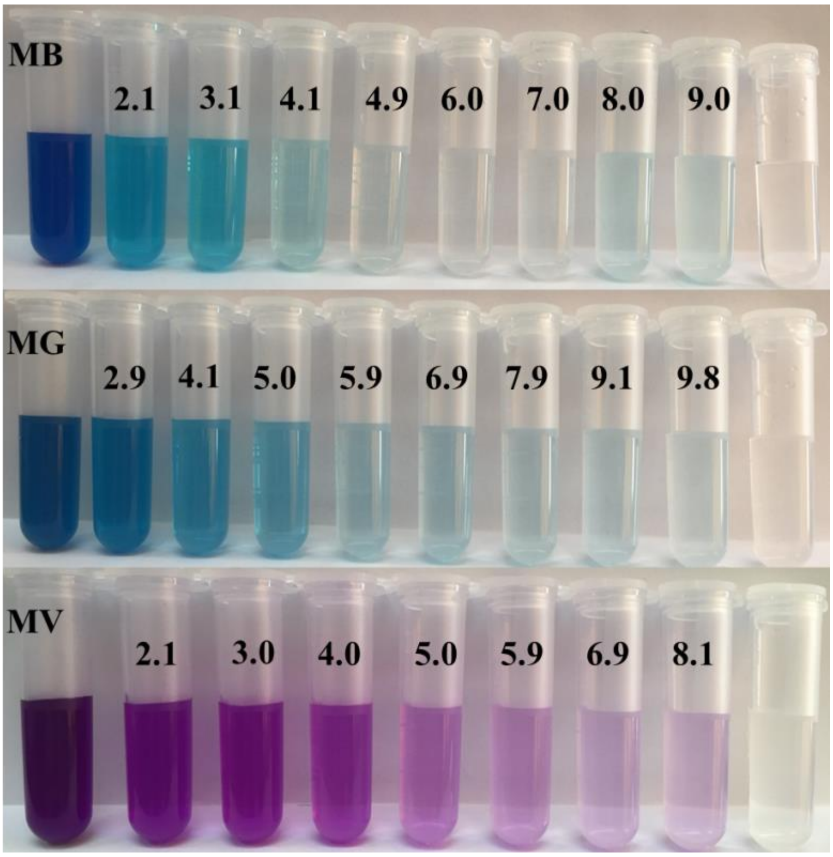

Figure S3. The images of wastewater before and after the removal of MB, MG and MV by the adsorbent with different pH (pH = 2.1-9.0 for MB, 2.9-9.8 for MG and 2.1-8.1 for MV, C_0 = 40 mg L^{-1}, V = 10 mL, temperature was 298 K, the right samples were water).

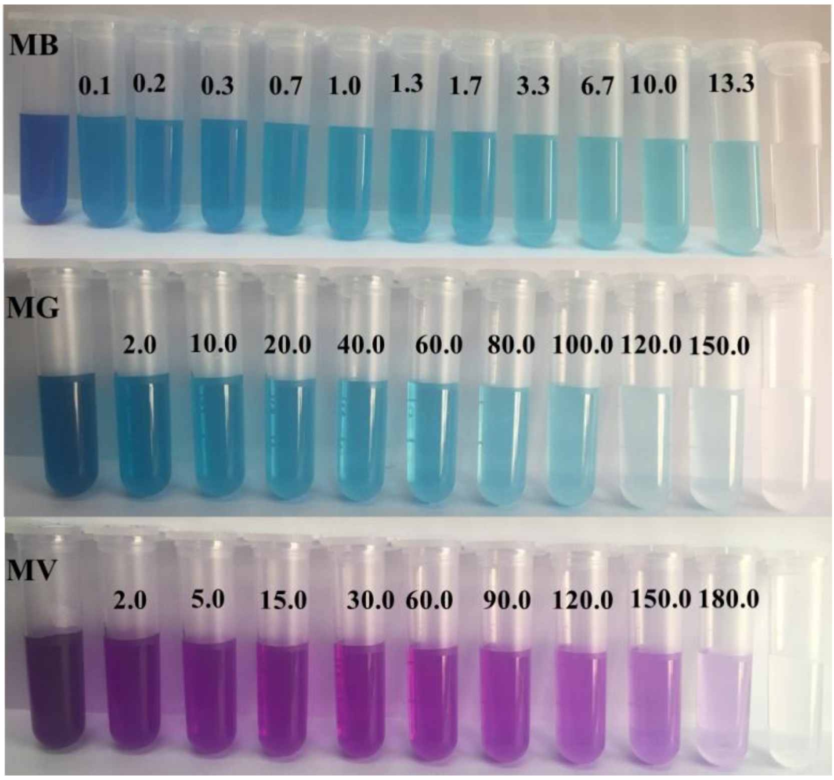

Figure S4. The images of wastewater before and after the removal of MB, MG and MV by the adsorbent with different contact time (contact time was 0.1-13.3 min for MB, 2-150 min for MG and 2-180 min for MV, C_0 = 40 mg L^{-1}, V = 10 mL, temperature was 298 K, the right samples were water).

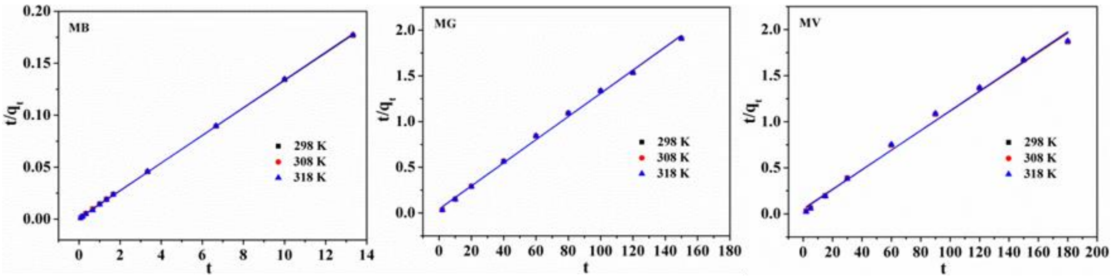

Figure S5. Pseudo-second-order kinetics for adsorption of MB and MG (m = 5 mg, C_0 = 40 mg L^{-1}, V = 10 mL, pH = 6, temperature at 298 K, 308 K and 318 K), MV (m = 4 mg, C_0 = 40 mg L^{-1}, V = 10 mL, pH = 6, temperature at 298 K, 308 K and 318 K).

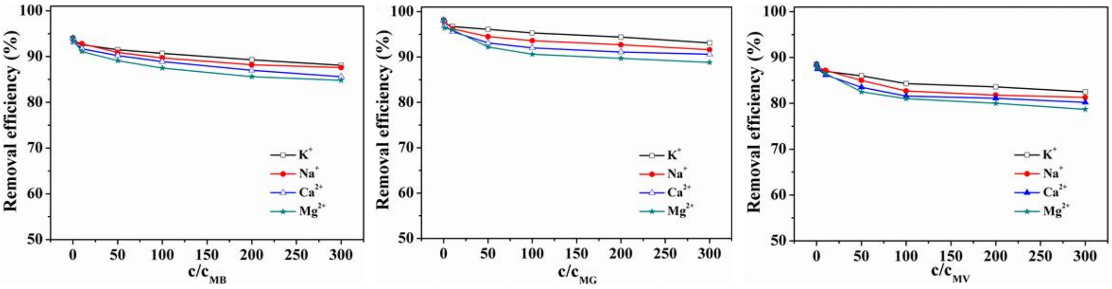

Figure S6. Effect of coexisting ions on adsorption of MB (m = 5 mg, C_{MB} = 40 mg L^{-1}, V = 10 mL, pH = 6, contact time was 10 min, temperature was 298 K), MG (m = 5 mg, C_{MG} = 40 mg L^{-1}, V = 10 mL, pH = 6, contact time was 120 min, temperature was 298 K) and MV (m = 4 mg, C_{MV} = 40 mg L^{-1}, V = 10 mL, pH = 6, contact time was 120 min, temperature was 298 K). The concentration ratio of coexisting ions (K^+, Na^+, Ca^{2+} or Mg^{2+}) to MB (c/c_{MB}), MG (c/c_{MG}) or MV (c/c_{MV}) was 0, 1, 10, 50, 100, 200 and 300, respectively.

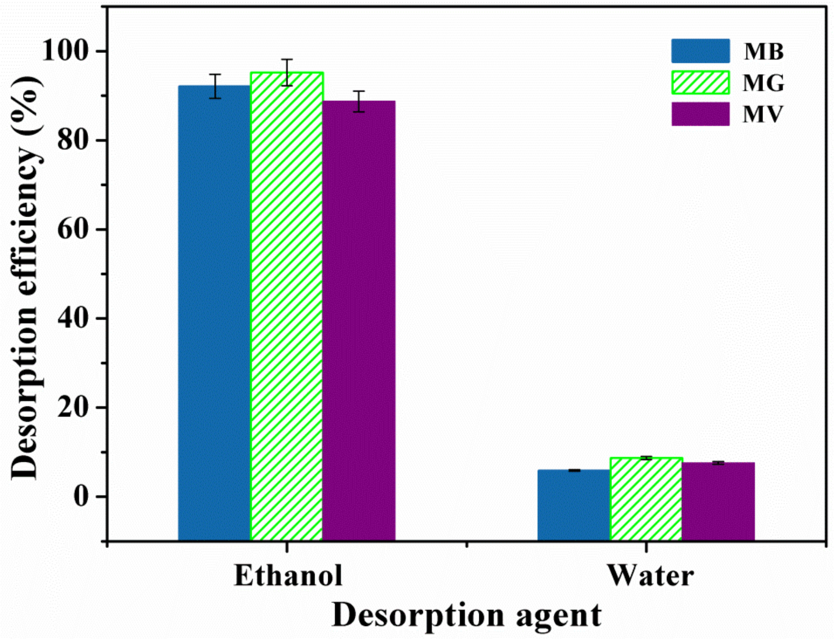

Figure S7. Desorption of MB (m = 5 mg, C_0 = 40 mg L $^{-1}$, V = 10 mL, pH = 6, contact time was 10 min, temperature was 298 K), MG (m = 5 mg, C_0 = 40 mg L^{-1}, V = 10 mL, pH = 6, contact time was 120 min, temperature was 298 K) and MV (m = 4 mg, C_0 = 40 mg L^{-1}, V = 10 mL, pH = 6, contact time was 120 min, temperature was 298 K) by using ethanol and water as desorption agent. Error bar = SD (n = 2).

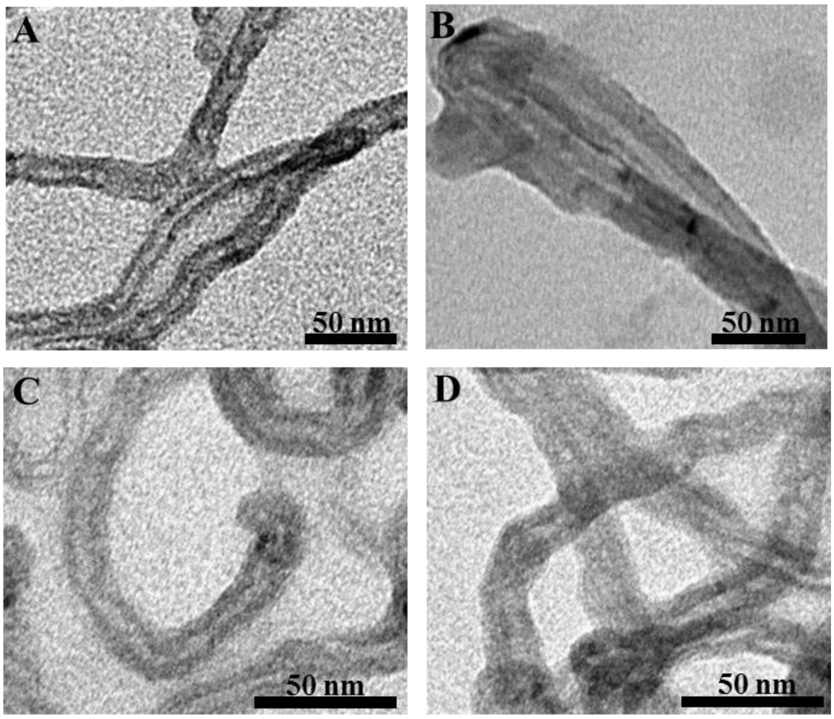

Figure S8. The TEM images of WHPA-OMCNT adsorbents before (A) and after (B) MB dye removal, the first (C) and the fifth (D) recycled ones (m = 5 mg, C_0 = 40 mg L $^{-1}$, V = 10 mL, pH = 6, contact time was 10 min, temperature was 298 K).

Table S1. Constants and correlation coefficients for the kinetic models of MB, MG and MV onto WHPA-OMCNT.

Kinetic model	Parameter	MB	R^2	MG	R^2	MV	R^2
Pseudo-first-order	k_1 (mg min g^{-1})<br>q_e (mg g^{-1})	0.1475<br>11.87	0.9279	0.0109<br>23.79	0.8699	0.0089<br>40.91	0.6244
Pseudo-second-order	k_2 (mg min g^{-1})<br>q_e (mg g^{-1})	0.1522<br>75.59	0.9999	0.0036<br>79.11	0.9976	0.0020<br>94.43	0.9934
Intraparticle diffusion	k_{dif} (mg g^{-1} min$^{-1/2}$)	4.6683	0.7548	1.7759	0.8962	2.2262	0.8935
Bangham	m<br>k_b (mg g^{-1})	18.7512<br>117.8685	0.9444	13.9782<br>54.4918	0.9644	12.6727<br>60.4889	0.8853

Table S2. Constants and correlation coefficients for pseudo-second-order kinetic models of MB, MG and MV onto WHPA-OMCNT at 298 K, 308 K and 318 K.

Temperature	Parameter	MB	R^2	MG	R^2	MV	R^2
298 K	k_2 (mg min g^{-1})	0.1522		0.0036		0.0020	
	q_e (mg g^{-1})	75.59	0.9999	79.11	0.9976	94.43	0.9934
	h (mg g^{-1} min^{-1})	869.6		22.6		17.8	
308 K	k_2 (mg min g^{-1})	0.1928		0.0037		0.0022	
	q_e (mg g^{-1})	75.30	0.9999	78.99	0.9975	93.90	0.9931
	h (mg g^{-1} min^{-1})	1093.4		23.5		19.0	
318 K	k_2 (mg min g^{-1})	0.2639		0.0039		0.0024	
	q_e (mg g^{-1})	75.02	0.9998	78.80	0.9975	93.37	0.9926
	h (mg g^{-1} min^{-1})	1485.2		24.3		20.7	

Table S3. Adsorption isotherm parameters for MB, MG and MV adsorption onto WHPA-OMCNT at 298 K, 308 K, 318 K.

Temperature	Adsorption isotherm	Parameter	MB	R^2	MG	R^2	MV	R^2
298 K	Henry	K_H	1.4923	0.7554	1.6359	0.5252	1.5303	0.8217
	Langmuir	b (L mg^{-1})<br>q_m (mg g^{-1})	0.0221<br>800.00	0.9644	0.1118<br>840.34	0.9989	0.0125<br>970.87	0.9888
	Freundlich	K_F<br>$1/n$	77.90<br>0.3810	0.9867	141.59<br>0.3647	0.8396	43.30<br>0.5171	0.9628
	D–R	β (mol^2 kj^{-2})<br>q_m (mg g^{-1})	4.6×10^{-7}<br>350.26	0.4353	4.2×10^{-7}<br>438.54	0.6599	1.0×10^{-5}<br>411.74	0.5470
308 K	Henry	K_H	1.2194	0.6964	1.5521	0.5128	1.1676	0.8197
	Langmuir	b (L mg^{-1})<br>q_m (mg g^{-1})	0.0185<br>735.29	0.9766	0.0881<br>826.45	0.9994	0.0124<br>826.45	0.9785
	Freundlich	K_F<br>$1/n$	57.76<br>0.4171	0.9474	120.44<br>0.3910	0.8243	45.00<br>0.4712	0.9641
	D–R	β (mol^2 kj^{-2})<br>q_m (mg g^{-1})	6.3×10^{-7}<br>312.95	0.3022	6.2×10^{-7}<br>417.03	0.6044	7.9×10^{-6}<br>368.43	0.5138
318 K	Henry	K_H	1.1315	0.7481	1.4595	0.5132	0.7857	0.8069
	Langmuir	b (L mg^{-1})<br>q_m (mg g^{-1})	0.0161<br>680.27	0.9686	0.0797<br>806.45	0.9991	0.0106<br>641.03	0.9806
	Freundlich	K_F<br>$1/n$	52.43<br>0.4109	0.9507	113.15<br>0.3939	0.8119	36.40<br>0.4494	0.9670
	D–R	β (mol^2 kj^{-2})<br>q_m (mg g^{-1})	7.5×10^{-7}<br>292.93	0.7059	8.3×10^{-7}<br>421.53	0.6401	8.9×10^{-6}<br>296.95	0.4345

Table S4. Thermodynamic parameters for MB, MG and MV adsorption onto WHPA-OMCNT.

Pollutants	Temperature	lnK_d	ΔH (kJ mol^{-1})	ΔS (J mol^{-1} K^{-1})	ΔG (kJ mol^{-1})
	298 K	1.401464			-3.430
MB	308 K	1.230555	-9.049	-18.857	-3.241
	318 K	1.172888			-3.053
	298 K	1.696313			-4.179
MG	308 K	1.601244	-5.046	-2.090	-4.150
	318 K	1.568847			-4.121
	298 K	1.075999			-2.716
MV	308 K	1.065459	-5.540	-9.476	-2.621
	318 K	0.934013			-2.527

Table S5. Asorption capacities of various adsorbents for MB, MG and MV.

Material	Target element	Isotherm	q_m (mg g^{-1})	Ref.
WHPA-OMCNT	MB<br>MG<br>MV	Langmuir	800.0<br>840.3<br>970.9	This paper
M-MWCNTs	MB	Langmuir	48.1	1
Fe_3O_4/SiO_2/HPG-COOH	MB	Langmuir	244.0	2
Carbon nanotubes	MB	Langmuir	35.0	3
Carbon monolith	MB	Langmuir	127.5	4
Cellulose/CNTs hybrid beads	MB	Langmuir	308.0	5
Fe_3O_4@POSS-SH	MG	Langmuir	111.0	6
Activated carbon	MG	Langmuir	4.3	7
Aminopropyl functionalized magnesium phyllosilicate	MG	Langmuir	130.6	8
Chitin hydrogels	MG	Langmuir	33.6	9
PAA/SiO_2 membranes	MG	Langmuir	220.5	10
h-XG/SiO_2	MV	Langmuir	378.8	11
Fe_3O_4/SiO_2/HPG-COOH	MV	Langmuir	244.0	2
3D graphenen oxide	MV	Langmuir	467.0	12
Magnetic multi-walled carbon nanotubes	MV	Langmuir	277.7	13
Sunflower seed hull	MV	Freundlich	92.6	14

1. Ai L.H. et al. Removal of methylene blue from aqueous solution with magnetite loaded multi-wall carbon nanotube: Kinetic, isotherm and mechanism analysis. *J. Hazard. Mater.* **198**, 282-290 (2012).

2. Zhou L., Gao C. & Xu W.J. Magnetic dendritic materials for highly efficient adsorption of dyes and drugs. *ACS Appl. Mater. Interfaces* **2**, 1483-1491 (2010).

3. Yao Y., Xu F., Chen M., Xu Z. & Zhu Z. Adsorption behavior of methylene blue on carbon nanotubes. *Bioresource Technol.* **101**, 3040-3046 (2010).

4. He X.Y. et al. Adsorption and desorption of methylene blue on porous carbon monoliths and nanocrystalline cellulose. *ACS Appl. Mater. Interfaces* **5**, 8796-8804 (2013).

5. Deng C. et al. Fabrication of spherical cellulose/carbon tubes hybrid adsorbent anchored with welan gum polysaccharide and its potential in adsorbing methylene blue. *Chem. Eng. J.* **200-202**, 452-458 (2012).

6. He H.B. et al. Mesostructured nanomagnetic polyhedral oligomeric silsesquioxanes (poss) incorporated with dithiol organic anchors for multiple pollutants capturing in wastewater. *ACS Appl. Mater. Interfaces* **5**, 8058-8066 (2013).

7. Hajati S., Ghaedi M. & Yaghoubi S. Local, cheep and nontoxic activated carbon as efficient adsorbent for the simultaneous removal of cadmium ions and malachite green: Optimization by surface response methodology. *J. Ind. Eng. Chem.* **21**, 760-767 (2015).

8. Lee Y.-C., Kim E.J., Yang J.-W. & Shin H.-J. Removal of malachite green by adsorption and precipitation using aminopropyl functionalized magnesium

phyllosilicate. *J. Hazard. Mater.* **192**, 62-70 (2011).

9. Tang H., Zhou W.J. & Zhang L.N. Adsorption isotherms and kinetics studies of malachite green on chitin hydrogels. *J. Hazard. Mater.* **209-210**, 218-225 (2012).

10. Xu R., Jia M., Zhang Y. & Li F. Sorption of malachite green on vinyl-modified mesoporous poly(acrylic acid)/SiO_2 composite nanofiber membranes. *Micropor. Mesopor. Mater.* **149**, 111-118 (2012).

11. Ghorai S., Sarkar A., Raoufi M., Panda A.B., Schönherr H. & Pal S. Enhanced removal of methylene blue and methyl violet dyes from aqueous solution using a nanocomposite of hydrolyzed polyacrylamide grafted xanthan gum and incorporated nanosilica. *ACS Appl. Mater. Interfaces* **6**, 4766-4777 (2014).

12. Liu F., Chung S.Y., Oh G. & Seo T.S. Three-dimensional graphene oxide nanostructure for fast and efficient water-soluble dye removal. *ACS Appl. Mater. Interfaces* **4**, 922-927 (2012).

13. Madrakiana T., Afkhamia A., Ahmadia M. & Bagheri H. Removal of some cationic dyes from aqueous solutions using magnetic-modified multi-walled carbon nanotubes. *J. Hazard. Mater.* **196**, 109-114 (2011).

14. Hameed B.H. Equilibrium and kinetic studies of methyl violet sorption by agricultural waste. *J. Hazard. Mater.* **154**, 204-212 (2008).
